# In an Ovine Model of Polycystic Ovary Syndrome (PCOS) Prenatal Androgens Suppress Female Fetal Renal Gluconeogenesis

**DOI:** 10.1371/journal.pone.0132113

**Published:** 2015-07-06

**Authors:** Fiona Connolly, Michael T. Rae, Katharina Späth, Lyndsey Boswell, Alan S. McNeilly, W. Colin Duncan

**Affiliations:** 1 MRC Centre for Reproductive Health, the University of Edinburgh, Edinburgh, United Kingdom; 2 School of Health, Life and Social Sciences, Edinburgh Napier University, Edinburgh, United Kingdom; Xavier Bichat Medical School, INSERM-CNRS—Université Paris Diderot, FRANCE

## Abstract

Increased maternal androgen exposure during pregnancy programmes a polycystic ovary syndrome (PCOS)-like condition, with metabolic dysfunction, in adult female offspring. Other *in utero* exposures associated with the development of insulin resistance, such as intrauterine growth restriction and exposure to prenatal glucocorticoids, are associated with altered fetal gluconeogenesis. We therefore aimed to assess the effect of maternal androgenisation on the expression of PEPCK and G6PC in the ovine fetus. Pregnant Scottish Greyface sheep were treated with twice weekly testosterone propionate (TP; 100mg) or vehicle control from day 62 to day102 of gestation. At day 90 and day 112 fetal plasma and liver and kidney tissue was collected for analysis. PEPCK and G6PC expression were analysed by quantitative RT-PCR, immunohistochemistry and western blotting. PEPCK and G6PC were localised to fetal hepatocytes but maternal androgens had no effect on female or male fetuses. PEPCK and G6PC were also localised to the renal tubules and renal PEPCK (*P*<0.01) and G6PC (*P* = 0.057) were lower in females after prenatal androgenisation with no change in male fetuses. These tissue and sex specific observations could not be explained by alterations in fetal insulin or cortisol. The sexual dimorphism may be related to the increase in circulating estrogen (*P*<0.01) and testosterone (*P*<0.001) in females but not males. The tissue specific effects may be related to the increased expression of *ESR1* (*P*<0.01) and *AR* (*P*<0.05) in the kidney when compared to the fetal liver. After discontinuation of maternal androgenisation female fetal kidney *PEPCK* expression normalised. These data further highlight the fetal and sexual dimorphic effects of maternal androgenisation, an antecedent to adult disease and the plasticity of fetal development.

## Introduction

Polycystic ovary syndrome (PCOS) is one of the most common endocrinopathies, affecting approximately 6–10% of women in their reproductive years [1,2]. It has a heterogeneous phenotype with ovarian, hormonal and metabolic anomalies, associated with obesity, insulin resistance and abnormalities of glucose handling [3–5]. While it first presents clinically in adolescence [2] there is increasing evidence that PCOS has its origins in fetal life [6]. Animal modelling of PCOS, using prenatal androgenisation in multiple species, highlights that fetal changes are associated with an adult phenotype. Exposure to androgens during pregnancy promotes the development of a PCOS phenotype in offspring with key metabolic features.

Rhesus monkeys and sheep demonstrate alterations in glucose handling with hyperinsulinemia, altered pancreatic beta cell function [7–12] and hepatic steatosis [10]. Although much less of rodent development is intrauterine, rats and mice also show an increase in body mass, impaired glucose and insulin signaling [13–15] and hepatic steatosis [13]. It is likely that prenatal androgenisation experiments can inform us about common pathways involved in the fetal origins of PCOS.

We previously reported that prenatally androgenised ewes developed both pancreatic and hepatic aberrations in adulthood with an increased insulin secretion in response to glucose and histological signs of fatty liver [10,11]. Pancreatic alterations from *in utero* androgenisation were found to initiate during fetal life, with modified gene expression for pancreatic function and development [11]. Therefore, we suspected that the liver may be susceptible to changes during fetal life, which manifest in adulthood as altered hepatic function and increased fatty liver changes [10]. It is documented that hepatic androgen responsive genes, which have key metabolic roles, include phosphoenolpyruvate carboxykinase (PEPCK) [16]. PEPCK is a transcriptionally regulated gene responsible for an early, rate-limiting step in gluconeogenesis [17,18].

The gluconeogenic pathway is involved in the release of glucose into the circulation, which primarily occurs in the liver but can occur in the kidney [19]. Gluconeogenesis is redundant during times of high external glucose supply, and it is inhibited in response to increased insulin, through its suppression of the expression of the key gluconeogenic genes PEPCK and glucose-6-phosphatase (G6PC). PEPCK catalyses the conversion of oxaloacetic acid to phosphoenolpyruvate while G6PC catalyses the final gluconeogenic step of glucose-6-phosphate to free glucose. While insulin suppresses gluconeogenic activity, both glucagon released during fasting, and glucocorticoids enhance such activity to increase blood glucose concentrations [20,21]. As regulated fetal gluconeogenesis is important for normal growth and development we hypothesised that dysregulation during prenatal androgenisation may have a role in the prenatal programming of future metabolic dysfunction.

We therefore aimed to quantify hepatic and renal *PEPCK* and *G6PC* expression, and pathways regulating their transcription in fetuses collected at d90 gestation from pregnant ewes treated biweekly with testosterone from d62 gestation. This regimen has been shown to promote a PCOS-like condition with metabolic dysfunction in adult female offspring [10,11,22].

## Materials and Methods

### Ethical Statement

Studies were reviewed by University of Edinburgh Animal Research Ethics Committee and conducted under Project Licence approved by the UK Home Office.

### Animal Treatments

All animal experimentation was conducted under license from the UK Home Office after ethical review. Scottish Greyface ewes were fed to achieve a comparable body condition score prior to estrous cycle synchronisation and timed mating with Texel rams. Pregnant ewes received biweekly intramuscular injections of either vehicle control (C) or 100 mgs testosterone propionate (TP; AMS Biotechnology Ltd., Abingdon, UK) from day 62 of gestation. Pregnant ewes were euthanised and male (C = 12, TP = 14) and female (C = 6, TP = 8) fetuses collected at d90. In a follow-up cohort, pregnant ewes received biweekly injections of either C or 100 mgs TP from d62-d102 of gestation. Ewes were euthanised, under Schedule 1 using barbiturate overdose, at d112 and female fetuses collected (C = 9, TP = 4).

### Tissue Collection

Fetal plasma was collected and stored at -80°C for subsequent analysis. Representative liver and kidney biopsies were 1) fixed in Bouins solution before being transferred to 70% ethanol for subsequent paraffin wax embedding and 2) snap frozen and stored at -80°C for subsequent RNA and protein extraction for gene analysis and western blotting studies. Unfortunately technological limitations meant that accurate fetal tissue weights were not recorded in these experiments.

### Quantitative Real Time PCR

Quantitative real time PCR was performed with SYBR Green as described previously [22,23]. Forward and reverse primers (Table 1) were designed using Primer3 Input version 0.4 online software (http://frodo.wi.mit.edu) with DNA sequences obtained at Ensembl Genome Browser. To confirm the validity of the gene product in the sheep both conventional PCR and amplicon sequencing were performed. Primer specificity and efficacy for qRT-PCR was evaluated through generation of standard curves with serial dilutions of cDNA, a standard curve slope of approximately -3 was accepted as efficient, and melt-curve analysis was also performed. Real time PCR was performed in duplicate 10μl reactions, negative controls included in each run per gene consisted of a cDNA reaction without reverse transcriptase (RT–ve) and a reaction replacing cDNA with nuclease-free water (template–ve). The expression of the unknown target gene was analysed relative to *GAPDH* as an internal control, and quantified using the ΔCt method as described previously [10,22,23].

**Table 1:** List of the primer pairs used in the SYBR Green Quantitative real-time PCR.

Gene	Forward Sequence	Reverse Sequence	Product Size (bp)
***GAPDH***	GGCGTGAACCACGAGAAGTATAA	AAGCAGGGATGATGTTCTGG	229
***PEPCK***	AAAGAGATACGGTGCCCATC	ATGCCAATCTTGGACAGAGG	178
***G6PC***	GAATGTCTGCCTGTCACGAA	ATCCAATGGCGAAACTGAAC	179
***IR***	CACCATCACTCAGGGGAAAC	CAGGAGGTCTCGGAAGTCAG	247
***IRS1***	ATCATCAACCCCATCAGACG	GAGTTTGCCACTACCGCTCT	240
***ADCY5***	CGCTCGTCTTCCTCTTCATC	CACAAACACCACCAAGGTCA	113
***ADCY6***	CACCCTGCACTTGGTCTTG	GATGTAACCGCGGGTCTCT	172
***GR***	AAGTCATTGAACCCGAGGTG	ATGCCATGAGGAACATCCAT	207
***HSD11B1***	ATTCTTGGCCTCATCGACAC	TCCATGATCTTCCTTCCTGG	191
***HSD11B2***	TGTGCCAAGAGCACTACAGG	CTCTACATGTGCCCTGCTCA	120
***ERS1***	GAATCTGCCAAGGAGACTCG	CCTGACAGCTCTTCCTCCTG	187
***AR***	GCCCATCTTTCTGAATGTCC	CAAACACCATAAGCCCCATC	233

### Immunohistochemistry

Sections were cut to 5μm and mounted on permafrost slides prior to dewaxing and rehydration. Antigen retrieval was carried out in a decloaking chamber (Biocare Medical, Concord, CA, US) containing sodium citrate retrieval buffer (0.01M, pH 6.0), then washed before incubation with H_2_O_2_ for 10min and blocked with avidin and biotin (Vector Laboratories Ltd., Peterborough, UK). This was followed by a further blocking step with 20% normal goat serum/5% bovine serum albumin (BSA) before incubation of sides with primary antibody diluted in serum overnight at 4°C (Table 2). Slides were washed in phosphate buffered saline containing 1% Tween 20 (PBST) to remove residual antibody and incubated with biotinylated secondary antibody (Table 2) for 1h, again washed in PBST and followed by Vectastain ABC Elite tertiary complex (PK-1600 Series; Vector Laboratories, Peterborough, UK) incubation for 1hr. Binding was visualized with 3,3′-diaminobenzidine (Dako, Cambridge, UK) for 30s. Sections were counterstained with haematoxylin and mounted using pertex. Negative controls consisted either of primary antibody incubated with a blocking peptide or, in the absence of a specific blocking peptide, serum with nonspecific immunoglobulins of equivalent concentrations.

**Table 2:** Antibodies used in immunohistochemistry and immunofluorescence and their concentrations.

Antigen	Antibody (1° Ab)	Dilution	2° Ab	Detection
**PEPCK**	Polyclonal Rabbit (H-300; sc-32879) (Santa Cruz Biotechnology Inc)	1:100	GARB	DAB
**G6PC**	Polyclonal Rabbit (Santa Cruz Biotechnology Inc)	1:800	GARB	DAB
**ESR1**	Monoclonal Mouse (MCA1974S; PPg5/10) (Serotec, Oxford, UK)	1:30	GAMB	DAB
**AR**	Polyclonal Rabbit (N20; sc-816) (Santa Cruz Biotechnology Inc)	1:25	GARB	DAB
**PEPCK**	Polyclonal Rabbit (H-300; sc-32879) (Santa Cruz Biotechnology Inc)	1:1000	GARB (1:500)	Avidin alexiflure 488 (1:200)
**AR**	Polyclonal Rabbit (N20; sc-816) (Santa Cruz Biotechnology Inc)	1:500	GARP (1:200)	Tyramide Cy3 (1:50)

Secondary antibodies are goat anti-rabbit biotinylated (GARB), goat anti-mouse biotinylated (GAMB) and goat ant-rabbit peroxidase conjucated (GARP). Immunohistochemistry detection was with diaminobenzidine (DAB).

### Immunofluorescence

Immunofluorescence was used to co-localise androgen receptor (AR) with PEPCK. The immunohistochemistry protocol detailed above was followed until the point of secondary antibody incubation, where a peroxidase conjugated secondary antibody was applied for 1hr before incubation with labelled Tyramide (PerkinElmer Life and Analytical Sciences, Inc., Shelton, CT, US) for 10 min (Table 2). Antigen retrieval was repeated, using microwave assisted antigen retrieval, before applying the second primary antibody and the secondary antibody conjugated to biotin (Table 2). Avidin Alexa fluor (Molecular Probes, Paisley, UK) was utilised for detection. Slides were mounted using Permafluor (Immunotech, Marseille, France) and images captured using the LSM 710 Confocal microscope (Carl Zeiss, Hertfordshire, UK).

### Western Blotting

Protein was extracted from frozen fetal kidney using a lysis buffer composed of 15NP-40, 150nM NaCl, 5mM EDTA, 50mM Tris-HCL pH 8.0, and a proteinase inhibitor (1 tablet per 20ml of buffer; Roche diagnostics GmBH, Mannhein, Germany). Protein concentration was determined using the Bradford Assay (Bio-Rad Laboratories Ltd.) and 20μg of protein was electrophoresed (SDS_PAGE) using a 7.5% polyacrylamide gel. Proteins were then transferred to a Hybond-P PVDF membrane (GE Healthcare UK Ltd., Buckinghamshire, UK) and membranes subsequently blocked in milk substitute before probing with the anti-PEPCK antibody diluted at 1:2000 concentration for 1 hr. Secondary antibody, peroxidase-conjugated goat anti-rabbit (1:2000), was applied for one hour at room temperature, then after washes (PBST 2x 5min), primary antibody binding was visualised by enhanced chemiluminescence (GE Healthcare UK Ltd) and developed with photographic processor (SRX-101A, Konica Minolta, Medical Imaging Inc., NJ, USA). After stripping gels were reprobed with B actin primary antibody (1:60000) with secondary goat anti-rabbit conjugated to peroxidase (1:2000) and visualised as above.

### Hormone Analysis

Testosterone and estradiol were measured in extracted serum using an in-house radioimmunoassay (RIA) as previously described [22,24]. For testosterone a primary antibody (rabbit anti-testosterone, AMS Biotechnology, Oxfordshire, UK) and radio-labelled testosterone (AMS Biotechnology) were used while estradiol utilised an in house antibody (ASRM32) and in house estradiol tracer conjugated to HRP. For cortisol extraction was not required. A rabbit anti-cortisol (20-CR5; Fitzgerald, MA, USA) antibody and radio-labeled cortisol (AMS Biotechnology) were utilised for the cortisol assay. All samples were assayed in duplicate and, internal controls consisted of a non-specific binding control, tracer only control and high, medium and low controls. Insulin was measured using an ovine specific kit (80-INSOV-E01, ALPCO Diagnostics, Salem, NH, USA) following manufacturers protocol. Colourmetric measurement of absorbance was carried out on a ThermoMax Microplate Reader (Molecular Devices, CA, USA) at 450 nm. A cubic spline fit standard curve was generated to extrapolate insulin concentrations using SoftMax Pro Software (Molecular Devices). All intra and inter-assay CVs were <10%.

### Statistical analysis

Data were analysed using Graph Pad Prism version 5.0 (GraphPad Software, San Diego California USA). Data are presented as mean ± S.E.M, with values of *P*<0.05 considered statistically significant. For qRT-PCR analysis an unpaired two-tailed students t-test was used to compare C versus TP and also liver versus kidney expression, unless data were not normally distributed where a Mann Whitney test was employed. ANOVA, with Bonferroni pairwise comparison was used when more than two variables were examined.

## Results and Discussion

### Prenatal androgenisation does not alter gluconeogenic enzyme expression in the fetal liver

**Fig 1:**
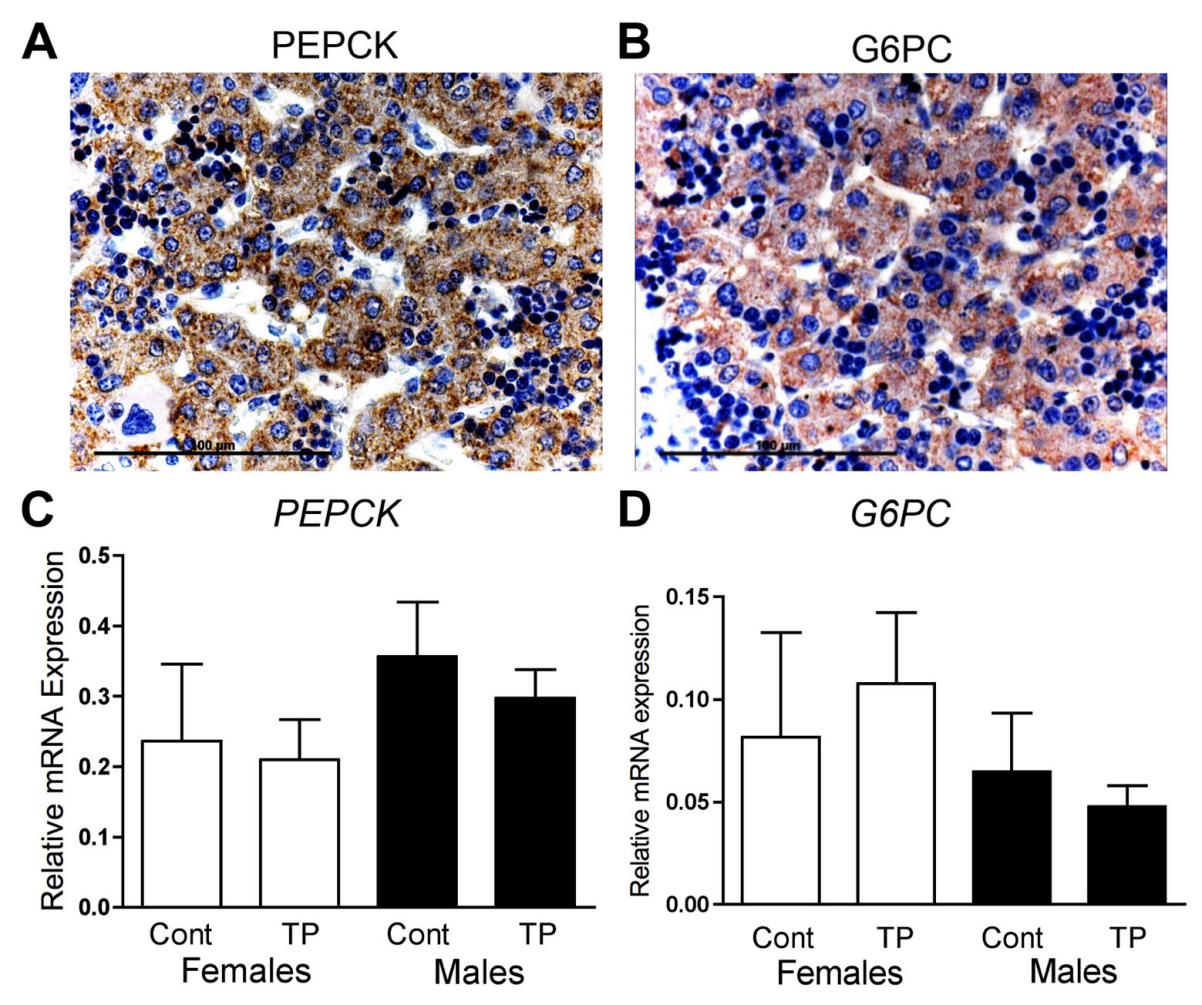
Fetal hepatic gluconeogenesis. Immunostaining of PEPCK (A) and G6PC (B) in the hepatocytes of the fetal liver (brown). Hepatic expression of *PEPCK* (C) and *G6PC* (D) quantified by qRT-PCR, in control (Cont) and prenatally androgenised females (TP; white bars) and males (black bars) at d90 of gestation. Values represent mean ±S.E.M. Scale bars represent 100μm.

Key enzymes in the gluconeogenic pathway, PEPCK and G6PC, could be localised to liver hepatocytes at d90 of fetal life (Fig 1A and 1B). As these genes are important determinants of fetal glucose homeostasis, which are transcriptionally regulated, qRT-PCR was performed to identify if prenatal androgen exposure altered their expression. Neither *PEPCK* (Fig 1C) nor *G6PC* (Fig 1D) were changed in response to prenatal androgen treatment in either the male or female ovine fetus.

### Prenatal androgenisation alters gluconeogenic enzyme expression in the fetal kidney

**Fig 2:**
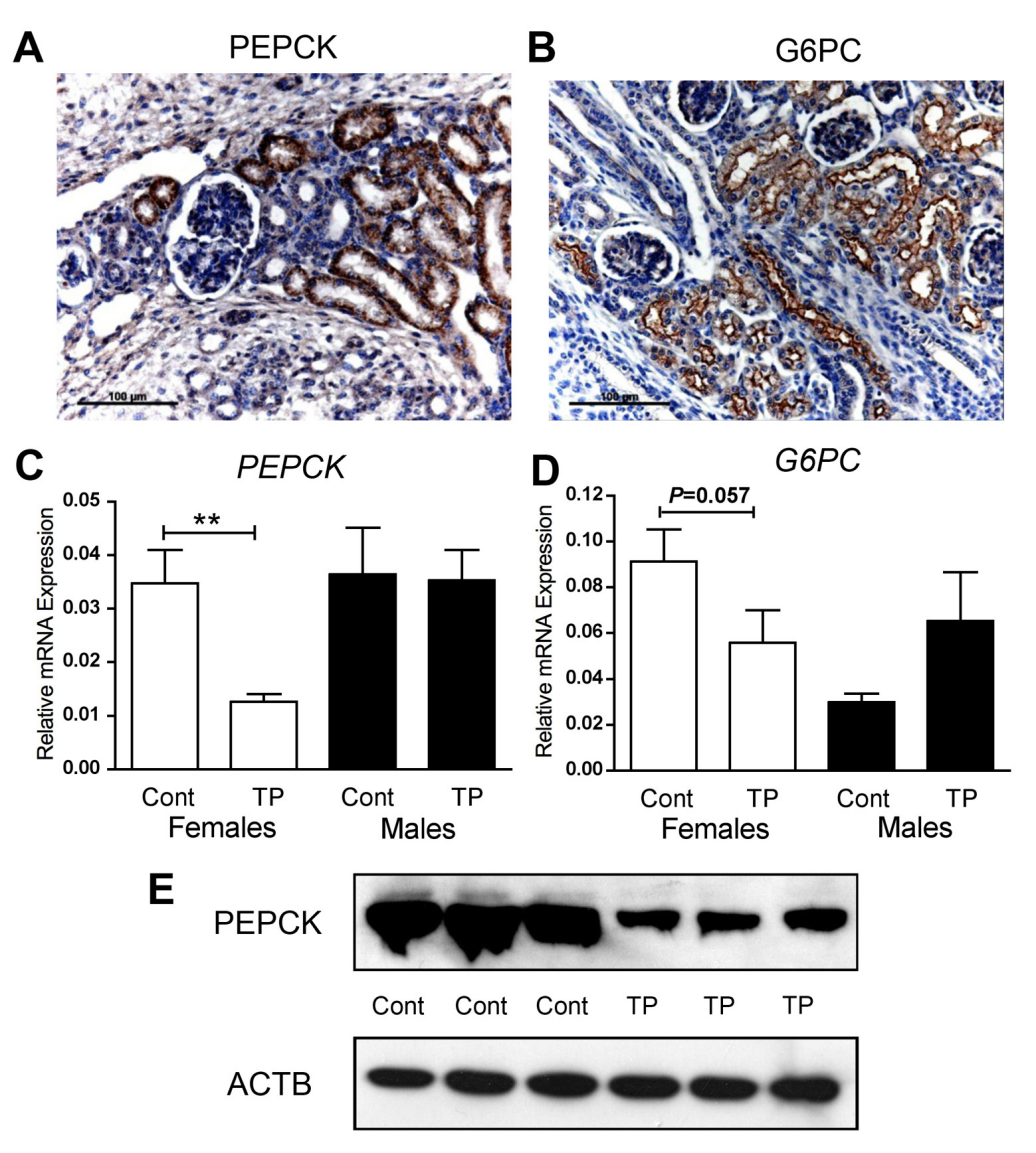
Fetal renal gluconeogenesis. Immunolocalisation of PEPCK (A) and G6PC (B) in the fetal kidney showing specific staining (brown) in the proximal tubules. Renal expression of *PEPCK* (C) and *G6PC* (D) quantified by qRT-PCR, of d90 females (white bars) and males (black bars) in both controls (Cont) and fetuses after maternal androgen exposure (TP). Representative western blot analysis of renal PEPCK in control (Cont) and prenatally TP exposed females and ACTB loading control (E). Values represent mean ±S.E.M. ** *P*<0.01. Scale bars represent 100μm.

As the kidney is the other main source of fetal gluconeogenic activity we investigated renal expression of PEPCK and G6PC. Both PEPCK (Fig 2A) and G6PC (Fig 2B) were localised to the proximal tubules in the outer cortex of the fetal kidney at d90 of fetal life. Renal expression of *PEPCK* was significantly reduced (*P*<0.05; Fig 2C) and *G6PC* expression showed a trend toward reduction (*P* = 0.056; Fig 2D) after prenatal androgenisation. This was however was only apparent in female fetuses and no such trend was noted in males (Fig 2C and 2D). This altered renal PEPCK expression was mirrored by protein expression (Fig 2E), as assessed by Western blotting. Therefore, prenatal treatment with TP induces a tissue and sex specific alteration of gluconeogenic enzyme expression in the ovine fetus.

### Insulin and glucagon action do not explain the differential effects on gluconeogenesis

**Fig 3:**
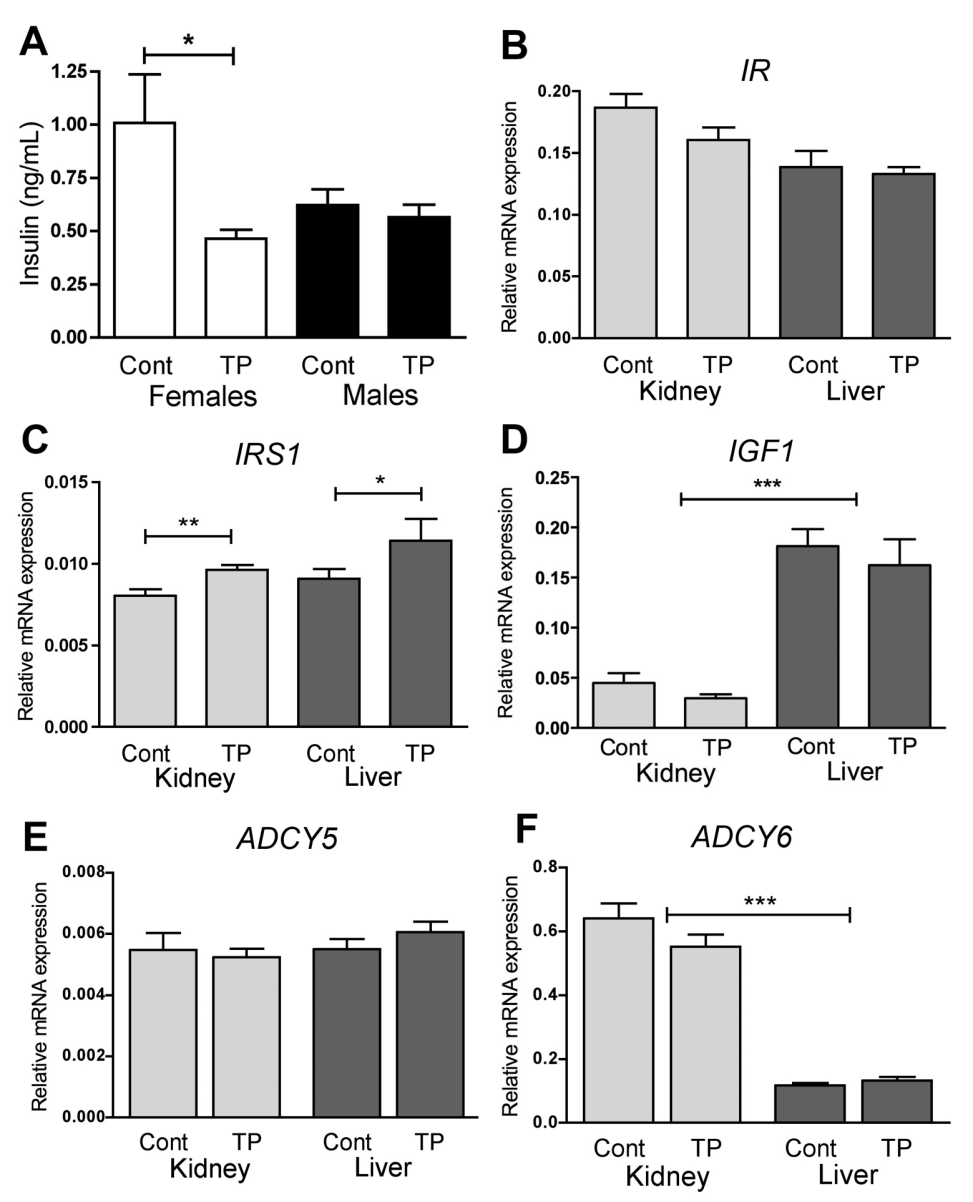
Role of insulin and glucagon. Serum insulin levels, measured by ELISA, in d90 female controls (Cont) and prenatally androgenised (TP) females (white) and males (black bars) (A). Modulators of insulin action; *IR* (B), *IRS1* (C), and *IGF1* (D) and measurement of glucagon signalling pathway *ADCY5* (E) and *ADCY6* (F) in the kidney (light bars) and liver (dark bars) quantified by qRT-PCR analysis, in d90 control (Cont) and TP exposed females. Values represent mean ±S.E.M.*P<0.05, ** *P*<0.01, *** *P*<0.001.

As insulin is a negative regulator of gluconeogenesis, fetal insulin concentrations were measured. In female fetuses circulating insulin was decreased by prenatal androgenisation while there were no differences in male fetuses (Fig 3A). Insulin could explain sexually dimorphic changes but a reduction in insulin would not be expected to drive a lower *PEPCK* expression. In females there was no change in *IR* (Fig 3B) expression but an increase in *IRS1* expression (*P*<0.01; Fig 3C) in the kidney. In the female liver the same increase in *IRS1* (*P*<0.05; Fig 3C) and lack of alteration of *IR* (Fig 3B) was also noted. There was no effect of *IGF1* expression, which was higher in the fetal liver than the kidney (Fig 3D). Glucagon works to increase PEPCK activity and ADCY5 and ADCY6 are downstream targets of the glucagon pathway. Prenatal androgenisation did not alter renal or hepatic expression of *ADCY5*, nor was there a tissue specific altered pattern of expression (Fig 3E). Although *ADCY6* had higher levels of expression in the fetal kidney than liver (*P*<0.001) its expression was not altered in response to prenatal androgen treatment (Fig 3F). Overall these results are not consistent with insulin or glucagon having a key role in the sex and tissue specific regulation of gluconeogenesis seen in prenatal androgenisation.

### Cortisol action does not explain the differential effects on gluconeogenesis

**Fig 4:**
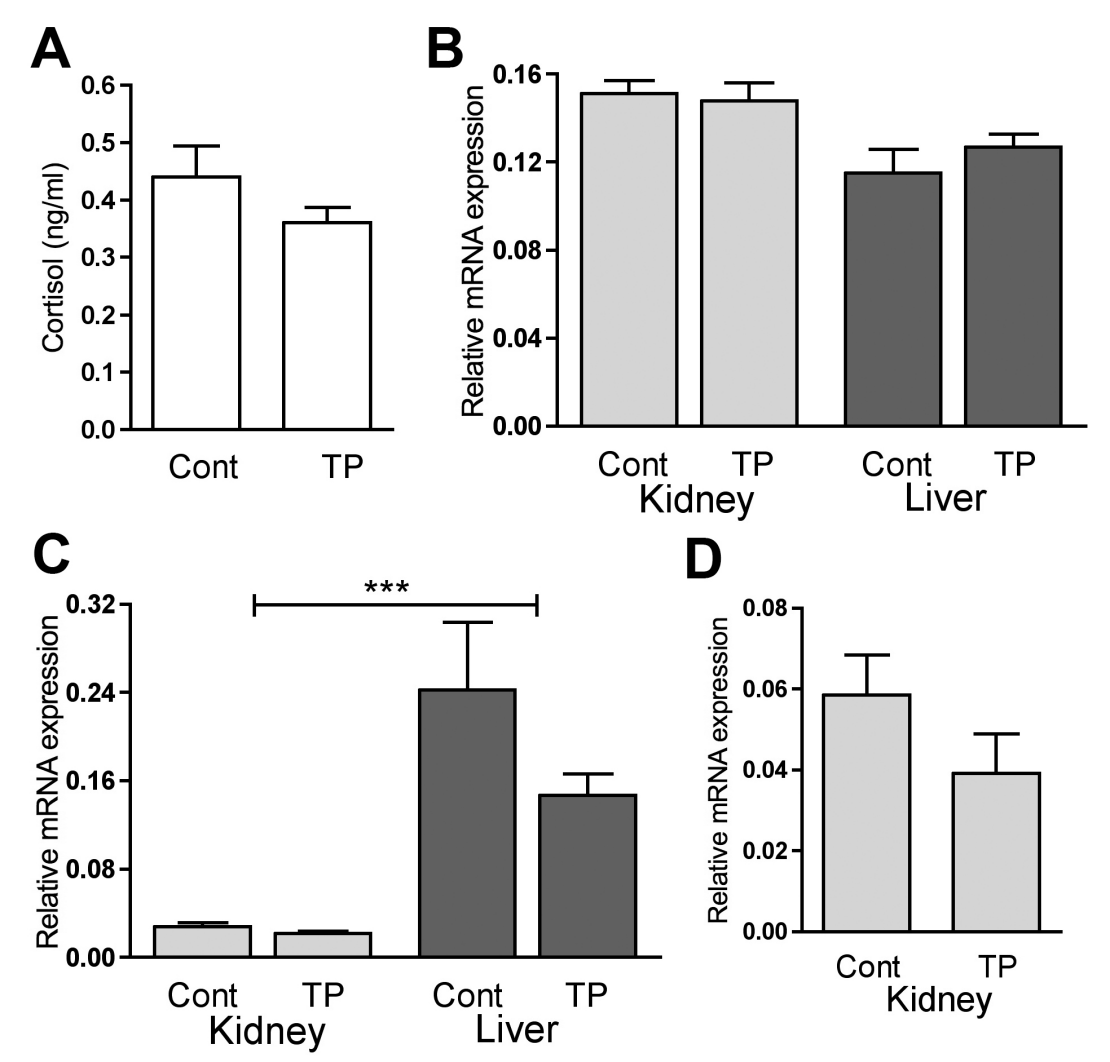
Role of cortisol. Serum cortisol levels in d90 females measured by RIA in controls (Cont) and fetuses collected after TP treatment (A). Renal (light bars) *GR* (B), *HSD11B1* (C) and *HSD11B2* (D) and hepatic (dark bars) *GR* (B) and *HSD11B1* (C) transcript abundance, quantified by qRT-PCR in the d90 female fetus. *HSD11B2* was not expressed in the fetal liver. Values represent mean ±S.E.M. *** *P*<0.001.

Cortisol is major positive regulator of gluconeogenesis. Circulating cortisol was not changed in female fetuses as a result of androgen exposure (Fig 4A). The glucocorticoid receptor was not altered in response to prenatal androgen treatment, nor did it have a tissue specific pattern of expression (Fig 4B). The peripheral cortisol generating enzyme *HSD11B1* was highly expressed in the liver compared to the kidney (*P*<0.001) and there was no change in response to androgenisation (Fig 4C). *HSD11B2* was not expressed in the liver and renal expression was also unaffected by TP treatment in d90 females (Fig 4D). This is not consistent with cortisol being involved in the differential regulation of gluconeogenesis after maternal androgenisation.

### Alterations in fetal sex hormones may explain the differential effects on steroidogenesis

**Fig 5:**
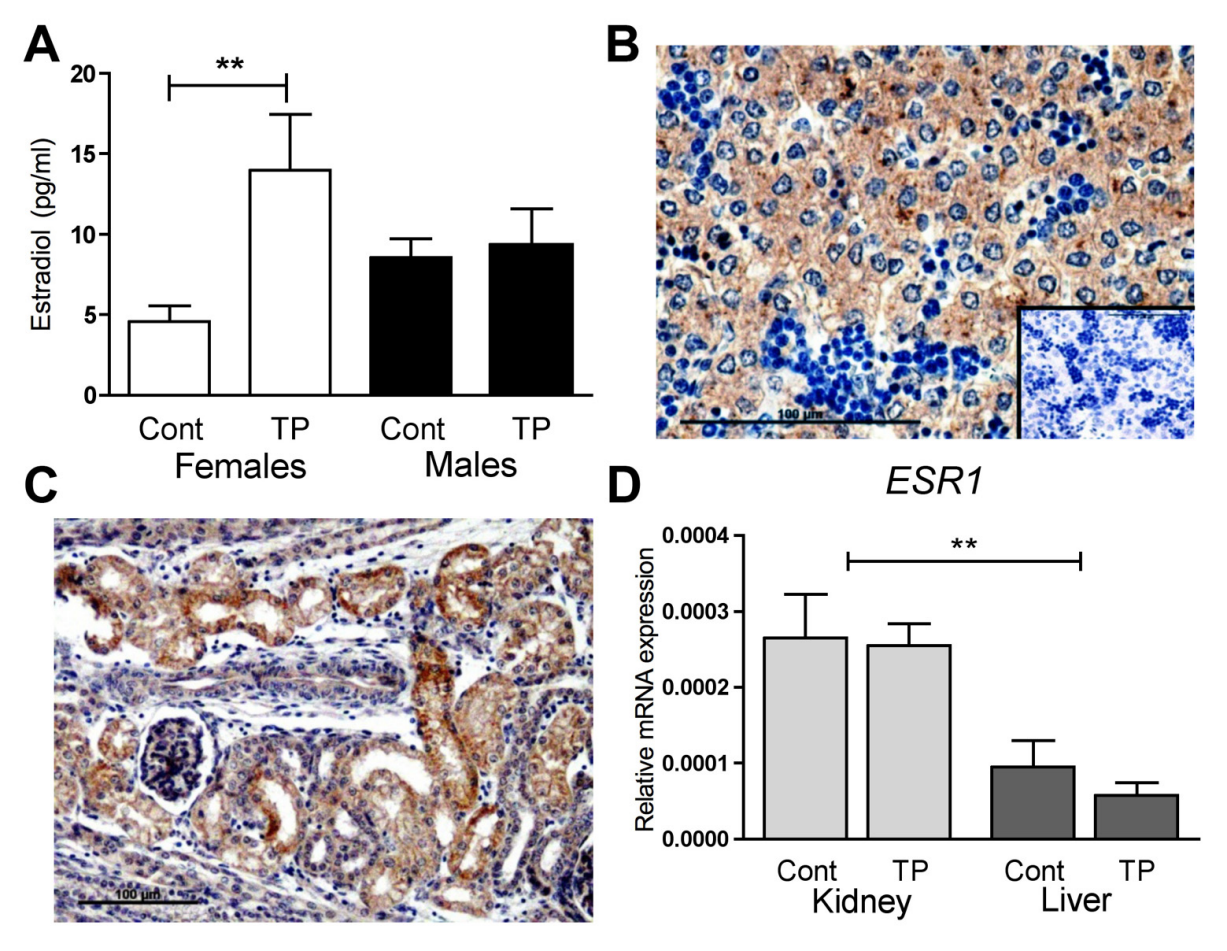
Role of estrogen. Circulating estradiol levels in control (Cont) females (white bars) and males (black bars) and those exposed to TP quantified through RIA (A). Immunolocalisation of ERS1 in a representative liver (B) and kidney (C) in the d90 female fetus. Inset is negative control. Renal (light bars) and hepatic (dark bars) *ERS1* expression in d90 females, whose mothers were prenatally treated with control oil (Cont) or TP (D). Values represent mean ±S.E.M. ** *P*<0.01. Scale bars represent 100μm.

**Fig 6:**
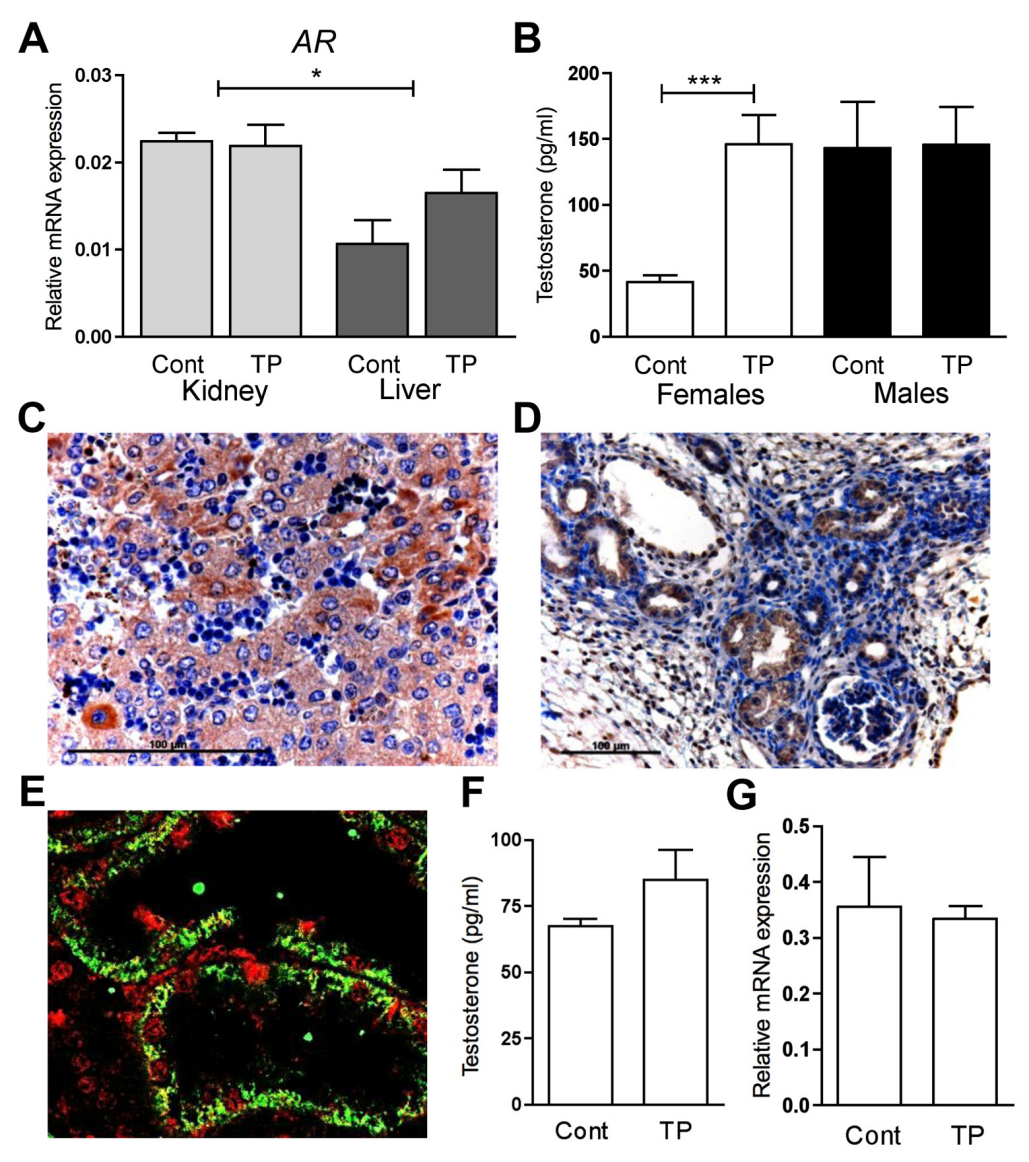
Role of Androgen. Transcript abundance for *AR* in the kidney (light bars) and liver (dark bars) of d90 females, treated prenatally with control (Cont) or TP quantified by qRT-PCR (A). Circulating serum testosterone levels in vehicle control (Cont) and TP exposed d90 females (white bars) and males (black bars) as measured by RIA (B). Androgen receptor localisation (brown) in the liver (C) and kidney (D) and co-localisation (red) with PEPCK (green) in the d90 fetal kidney at higher magnification (E). Circulating serum testosterone levels measured by RIA, in d112 females, prenatally exposed to C (Cont) or TP from d62-102 of ovine gestation (F). Renal *PEPCK* expression in females from the same treatment regime, controls (Cont) and TP treatment (G). Values represent mean ±S.E.M. ** *P*<0.01, *** *P*<0.001 Scale bars represent 100μm.

Fetal estradiol concentrations are increased after maternal androgenisation in the female fetus (*P*<0.01) but not the male fetus (Fig 5A). ESR1 could be detected at low levels in liver cells (Fig 5B) and it was specifically localised to the tubules in the fetal renal cortex (Fig 5C). *ESR1* was expressed in higher levels in the female fetal kidney than the liver (*P*<0.05; Fig 5D) and there was no effect of prenatal androgenisation. However, gene expression for *ESR1* was low and at the limits of assay detection. *AR* however was robustly expressed and at a greater level in the fetal kidney than the fetal liver (*P*<0.01; Fig 6A). In addition, as reported previously [24], maternal androgenisation increased female fetal androgen concentrations (*P*<0.001) while having no effect on male fetal androgens (Fig 6B). Little AR could be detected in the nuclei of hepatocytes (Fig 6C) while it was detected in the nuclei of fetal renal tubules (Fig 6D). Renal tubular cells synthesising PEPCK also expressed androgen receptors (Fig 6E). In addition after stopping maternal androgenisation female fetal testosterone concentrations normalised (Fig 6F) as did renal *PEPCK* expression (Fig 6G). This means that direct alterations in sex steroid action, particularly androgens, may explain both the tissue and sex specific effect of androgenisation on fetal gluconeogenesis.

## Discussion

In this study we found no difference in the expression of *PEPCK* in fetal liver after prenatal androgenisation. However we found that fetal renal *PEPCK* expression was significantly reduced at the time of maternal androgenisation in female fetuses but not in male fetuses. Further investigation of this tissue and sexual dimorphism suggested that this was a consequence of differential fetal and tissue androgen exposure and action. This effect is plastic as renal *PEPCK* expression normalised after maternal androgenisation ceased. Maternal androgenisation is associated with abnormalities of glucose handling in adult female offspring. This study further suggests that there are differences in glucose handling *in utero*.

Adult pancreatic structure and function is altered in female adult offspring by prenatal androgenisation and this has predictive fetal antecedents [11]. As liver structure and function is also altered in female adult offspring with increased fatty liver and augmented transcripts of *IGF1*, *AR* and *GR* [10] we investigated candidate fetal antecedents. Fetal gluconeogenesis has an important role in fetal life with regards to maintaining glucose supply, gluconeogenic enzymes are localised to hepatocytes [25], and it is a candidate pathway involved in the fetal programming of adult metabolic disease [26,27]. In addition, hepatic gluconeogenesis is altered in type 2 diabetes mellitus and increased in cases of fatty liver disease [21,28]. Intrauterine growth restriction (IUGR) increased the risk of adult metabolic diseases and diabetes and sheep fetuses with IUGR had increased hepatic *PEPCK* and *G6PC* [27]. Prenatal androgenisation however is not associated with alterations in fetal hepatic gluconeogenic enzyme expression.

The kidney is also a site of gluconeogenesis [19]. Indeed liver specific G6PC knockout mice are able to fully sustain glucose levels during fasting [29]. There is also some evidence that renal gluconeogenesis might be more important in the fetus than in the adult [30]. In the sheep fetus there is at least as much gluconeogenic activity in the kidney as in the liver [31]. In accordance with previous studies we immunolocalised both PEPCK and G6PC to the proximal tubules of the kidney cortex [25]. In addition, we found a female specific decrease in both renal *PEPCK* and *G6PC* abundance in female fetuses, as a result of prenatal androgenisation, which did not occur in males. Therefore prenatal androgenisation induced a tissue specific change to gluconeogenic gene expression as well as a sexual dimorphic pattern of alteration.

It is uncertain what effect this has on fetal glucose concentrations. It is felt that endogenous glucose production is minimal through most of gestation and that the main role of fetal glucose production is in later gestation in the presence of poor placental function, starvation or parturition [31]. Certainly there is a gestationally dependent increase in fetal gluconeogenic enzyme expression [31,32]. In this study we did not collect suitable samples for reliable fetal glucose estimation. The lambs born after prenatal androgenisation have no differences in birthweight or growth trajectory in the first year of life despite developing insulin resistance during this time [10]. The altered expression of renal PEPCK however suggests antecedent alterations of glucose handling induced by maternal androgen exposure in the mid-gestation female fetus.

One interesting observation was the tissue specific effect. It is known that maternal androgenisation can increase maternal glucose concentrations and these will pass into the fetus [33]. This may be a stimulus to reduce fetal gluconeogenic requirements but is it not clear why such an effect would be tissue or sex specific. As gluconeogenesis is regulated by insulin and glucocorticoids, which act by regulating the transcription of gluconeogenic enzymes, we investigated the pathways involved in regulation of gluconeogenesis.

Insulin inhibits *PEPCK* gene transcription to modulate glucose levels in the blood [21,34]. We found circulating insulin levels were decreased in the TP treated fetal females, which may be explained by alterations to the fetal pancreas [11] but would not suggest a rationale for an increase in *PEPCK* expression. Investigation into downstream modulators of insulin action in the kidney and liver found that *IRS1* expression was increased in response to TP that is potentially a compensatory mechanism for the reduction in circulating insulin levels. In addition IGF1 can mimic the actions of insulin, including inhibition of gluconeogenesis [35] and adult hepatic IGF-1 is increased by prenatal androgenisation [10]. However we found no differences in fetal *IGF1* expression in the liver or kidney in prenatally androgenised females. Indeed insulin was unable to suppress the increased gluconeogenesis induced by IUGR in the ovine fetus [27]. While there are sex specific effects on circulating insulin this does not seem to explain the sexual and tissue dimorphism in *PEPCK* expression in response to prenatal TP excess.

Cortisol acts as a positive regulator of gluconeogenesis through increasing activity of gluconeogenic enzymes [34]. There is a coordinated increase in cortisol levels with gluconeogenic enzymes in the sheep fetus during gestation [32]. Fetal adrenalectomy prevented the rise in gluconeogenic enzymes suggesting a role for cortisol in the regulation in fetal gluconeogenesis [32]. In fetal sheep with IUGR fetal cortisol was correlated with glucose production rates [27] Administration of dexamethasone in late gestation increased fetal G6PC activity in the liver and kidney of ovine fetuses [36] and this might be mediated by positive effects of glucocorticoids on fetal thyroid function [31]. No change was noted in female fetal cortisol concentrations in response to prenatal androgenisation, nor was there a treatment specific alteration to GR. While cortisol has a clear role in the maturational regulation of fetal gluconeogenesis it does not explain the effect of prenatal androgenisation.

11βHSD type 1 (encoded by HSD11B1) metabolises cortisone (inactive) to cortisol whereas 11βHSD type 2 (encoded by HSD11B2) directs the opposing dehydrogenation reaction, thus decreasing cortisol action [37]. The kidney expresses high levels of HSD11B2 that could explain tissue specific effects of cortisol. Indeed fetal adrenalectomy altered hepatic gluconeogenesis without effecting renal gluconeogenesis [32]. However there was no effect of androgenisation on the expression of enzymes regulating local cortisol availability. Differential cortisol effects seem like an unconvincing explanation for the altered gene expression found in the key gluconeogenic enzymes induced by prenatal androgenisation.

There is evidence for sexual dimorphism in gluconeogenesis. Low protein diet during pregnancy markedly induced fetal gluconeogenic enzyme expression in rodent male fetuses rather than female fetuses [38]. This suggests that sex steroids may have a role in the regulation of fetal gluconeogenesis. It is clear that females have increased circulating estrogen and androgen concentrations after maternal androgenisation while males do not. This seems to be related to the plasticity in the fetal testis for the modulation of endogenous hormone production using biofeedback [24]. Indeed, it has been previously shown that *PEPCK* is significantly decreased in response to testosterone, estrogen and a combination of both [16]. This suggests that sex steroids could be involved in the sexual dimorphic alterations in fetal gluconeogenesis.

As fetal sex steroids circulate, it was not clear why the fetal kidney may be more sensitive to alterations in sex steroid-regulation of gluconeogenesis than the liver. Hepatic immunostaining of ESR1 protein displayed a cytosolic location rather than nuclear, in contrast to renal ESR1 where nuclear localisation is evident. In addition renal estrogen receptor expression was increased when compared to that in the liver even although only a small proportion of renal cells express ESR1. However it is pertinent to note that expression levels are extremely low and although plausible altered estrogen may not be primary mechanistic explanation for our findings.

Androgens are attractive candidates for the sex and tissue specific changes. *AR* expression was tissue specific with lower hepatic expression compared to renal expression and AR, like ERα, did not show a nuclear localisation in the liver in contrast to that in the kidney. Cells expressing PEPCK in the fetal kidney co-express nuclear AR. Further evidence for a direct effect of testosterone was achieved when renal *PEPCK* was examined ten days post maternal androgen treatment and where circulating testosterone was not elevated [24]. In females at this time renal *PEPCK* expression had normalized and was comparable to controls. These observations would be consistent with a possible direct role for testosterone in negative regulation of fetal renal PEPCK.

In humans, female fetuses are exposed to variable androgen concentrations before birth. There is evidence that women with PCOS, and daughters of women with PCOS, experience more androgens before birth [39]. Whether this is genetic or environmental, or a combination of both, is not certain but knowledge of the effects of increased androgens in female fetuses may have clinical correlates in the developmental aetiology of PCOS. The study of the effects of androgens in female fetuses may increase our understanding of prenatal antecedents of metabolic dysfunction in women with PCOS.

## Conclusions

In conclusion, like previously reported studies from our and other laboratories, we demonstrate how females and males show a contrasting response to exogenous insults. The adult consequences of these specific alterations during key fetal growth are not known but it is evident that in adulthood female offspring exposed to androgens from d62 to d102 gestation have an adverse metabolic phenotype [10]. It is not clear if this alteration affects glucose availability or is involved in the development of later abnormalities of glucose handling. However this study signifies the potential for androgens to alter an important fetal metabolic pathway already implicated in the programming of adult health and disease.
